# Condensed Internet-delivered prolonged exposure provided soon after trauma: a randomised trial

**DOI:** 10.1017/S0033291721003706

**Published:** 2023-04

**Authors:** Maria Bragesjö, Filip K. Arnberg, Klara Olofsdotter Lauri, Kristina Aspvall, Josefin Särnholm, Erik Andersson

**Affiliations:** 1 Department of Clinical Neuroscience, Division of Psychology, Nobels väg 9, Karolinska Institutet, 171 77 Stockholm, Sweden; 2 Department of Neuroscience, Psychiatry, National Centre for Disaster Psychiatry, 751 24 Uppsala, Sweden; 3 Stress Research Institute, Stockholm University, 106 91 Stockholm, Sweden; 4 Department of Clinical Neuroscience, Centre for Psychiatry Research, Karolinska Institutet, 171 77 Stockholm, Sweden; 5 Stockholm Health Care Services, Region Stockholm, Stockholm, Sweden

**Keywords:** early intervention, post-traumatic stress, prolonged exposure, the Internet, trauma

## Abstract

**Background:**

Exposure to trauma is common and can have a profoundly negative impact on mental health. Interventions based on trauma-focused cognitive behavioural therapy have shown promising results to facilitate recovery. The current trial evaluated whether a novel, scalable and digital early version of the intervention, Condensed Internet-Delivered Prolonged Exposure (CIPE), is effective in reducing post-traumatic stress symptoms.

**Method:**

A single-site randomised controlled trial with self-referred adults (*N* = 102) exposed to trauma within the last 2 months. The participants were randomised to 3 weeks of CIPE or a waiting list (WL) for 7 weeks. Assessments were conducted at baseline, week 1–3 (primary endpoint), week 4–7 (secondary endpoint) and at 6-month follow-up. The primary outcome measure was PTSD Checklist for DSM-5 (PCL-5).

**Results:**

The main analysis according to the intention-to-treat principle indicated statistically significant reductions in symptoms of post-traumatic stress in the CIPE group as compared to the WL group. The between-group effect size was moderate at week 3 (bootstrapped *d* = 0.70; 95% CI 0.33–1.06) and large at week 7 (bootstrapped *d* = 0.83; 95% CI 0.46–1.19). Results in the intervention group were maintained at the 6-month follow-up. No severe adverse events were found.

**Conclusions:**

CIPE is a scalable intervention that may confer early benefits on post-traumatic stress symptoms in survivors of trauma. The next step is to compare this intervention to an active control group and also investigate its effects when implemented in regular care.

## Introduction

Exposure to trauma is a major public health problem. Population-based data from around the globe show an estimated life-time prevalence of exposure to psychologically traumatic events of 70% which may have a profoundly negative impact on mental health, leading to reactions as intrusions, avoidance, negative alterations in cognitions and mood, and hyperarousal (Koenen et al., 2017). These reactions are expected in the immediate aftermath, and, for many individuals, they fade with time. For those in whom the reactions persist and are disrupting within the first month following the event, the diagnosis of acute stress disorder can be used (American Psychiatric Association, 2013). In approximately 5–6% of those exposed to trauma, the reactions develop into long-term symptoms that fulfil the criteria for post-traumatic stress disorder (PTSD; Koenen et al., 2017). PTSD is a debilitating mental disorder in itself and is associated with increased risks for suicide, drug and alcohol dependence, sick leave, and several somatic and mental disorders (Kessler, Sonnega, Bromet, Hughes, & Nelson, 1995; McFarlane, Atchison, Rafalowicz, & Papay, 1994; Song et al., 2018).

One way to facilitate and speed up the recovery process could be to provide brief, easily scalable psychological interventions early after a traumatic event. Brief trauma-focused CBT (CBT-T) provided face-to-face during the first month after exposure to a traumatic event has been shown to be effective in reducing symptoms of post-traumatic stress (Bryant et al., 2008), and attempts have been made to provide CBT-T in the acute phase post-trauma (Bragesjö et al., 2020; Maples-Keller et al., 2020; Rothbaum et al., 2012). However, delivering trauma-focused therapy face-to-face by a trained therapist to the large populations of afflicted soon after trauma may be difficult to implement in many clinical settings; digital interventions could be a partial solution to this problem. A recent review found that digital CBT-T treatments for PTSD outperform waitlist controls; however, the certainty of the evidence was assessed as low, and most trials did not concern early interventions (Simon et al., 2021). In contrast, a brief, unguided, digital early intervention after trauma in a hospital emergency department was not found to be superior to a waitlist control in reducing symptoms of post-traumatic stress (Mouthaan et al., 2013). As a way to scale up the outreach of early interventions for individuals who have been exposed to trauma, our research group has developed and evaluated a digital intervention (Condensed Internet-delivered Prolonged Exposure; CIPE) in a pilot randomised trial. The rationale for CIPE was to provide a faster way to alleviate the symptoms of post-traumatic stress that the majority of trauma afflicted will experience and possibly prevent the subsequent development of PTSD and other comorbidities. Provided within the first 2 months after exposure to trauma, the intervention was shown to be feasible, acceptable and initially to reduce the symptoms of post-traumatic stress (Bragesjö, Arnberg, Särnholm, Olofsdotter Lauri, & Andersson, 2021*a*). The current trial aimed to assess the efficacy of CIPE as compared to the waiting list in a larger sample and with a longer controlled follow-up period.

## Method

### Trial design

The trial used a randomised controlled design comparing the intervention against a waiting list control condition. The pre-specified study plan is available at the Open Science Framework (https://osf.io/576xz). Participants were recruited nationwide in Sweden through self-referral. The trial was conducted at a single site (Karolinska Institutet, Stockholm, Sweden). We used a waiting list to control for the natural recovery of psychological distress after trauma. The process of natural recovery, seen in the majority of afflicted subjects, typically occurs within the first 3 months after exposure to a traumatic event (Bryant, 2003; Galea et al., 2002; Rothbaum, Foa, Riggs, Murdock, & Walsh, 1992) and we therefore decided that the participants should complete the intervention within that time frame. The primary endpoint was set to week 3 (intervention completion) and the secondary endpoint was set to week 7 (1-month follow-up). Participants randomised to the waiting list crossed over to the intervention after the controlled study period (week 7). Naturalistic long-term follow-ups were conducted at 6-month post-intervention. The National Ethical Review Board in Sweden approved the study (registration ID: 2019-04413). The trial was registered on ClinicalTrials.gov on 19 October 2019 before any participant was enrolled (registration ID: NCT03850639). The trial is reported in accordance to the CONSORT statement for non-pharmacological treatments.

### Participants

**Fig. 1.**
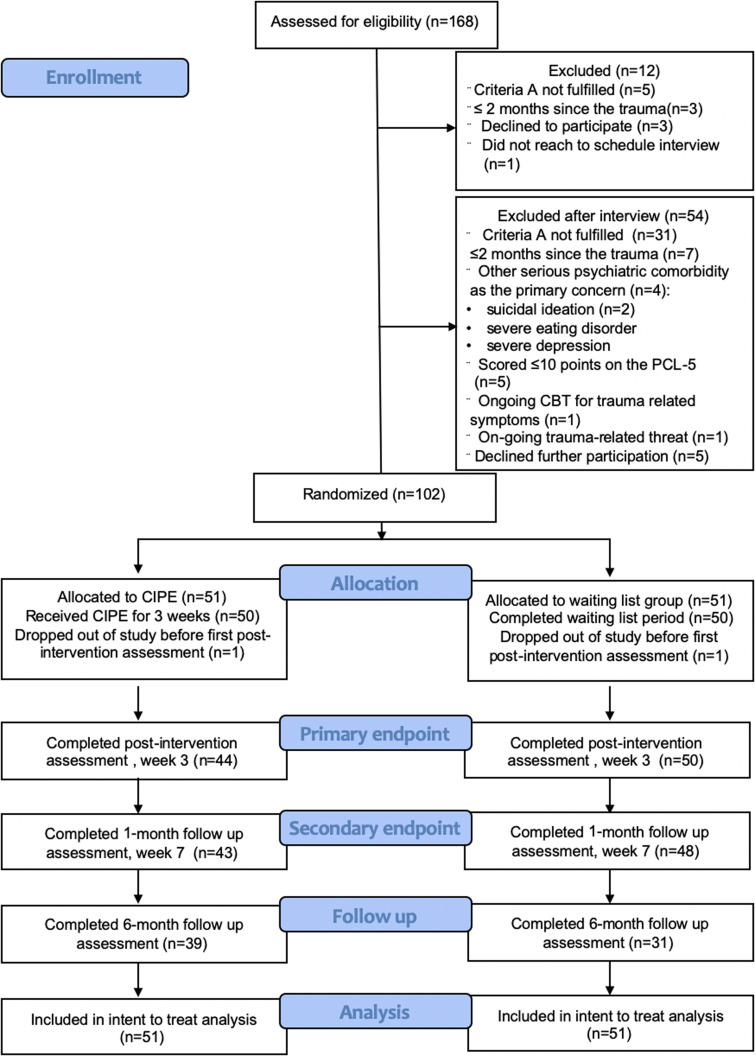
Trial participant flow.

The study was open to adult Swedish residents exposed to a traumatic event within 2 months prior to inclusion. Trauma was defined according to criterion A for PTSD in the fifth version of the Diagnostic and Statistical Manual of Mental Disorders (i.e. exposed to actual or threatened death, serious injury, or sexual violence; American Psychiatric Association, 2013). Participants had to have at least some symptoms of post-traumatic stress to be eligible for the trial; in this case, a total score of ⩾10 points on the PTSD Symptom Checklist for DSM-5 (PCL-5). According to the PCL-5 scoring interpretation from the National Centre of PTSD, a ⩾10 point reduction reflects a clinically meaningful change and we wanted to assure that each participant would be able to benefit from the treatment to that extent (Weathers et al., 2013). Exclusion criteria were: (a) other serious psychiatric comorbidity as the primary concern (e.g. on-going substance abuse, untreated bipolar disorder, psychotic symptoms, severe depression or high suicide risk) based on the assessor's clinical judgment after reviewing self-rating scales and conducting the Mini International Neuropsychiatric Interview (MINI; Sheehan et al., 1998); (b) currently receiving CBT for trauma-related distress; and (c) ongoing trauma-related threat (e.g. living with a violent spouse). Participants on psychotropic medication had to report a stable dose for 2 weeks prior to inclusion in the study. Excluded participants were given advice on how to seek regular mental health care. The participant flow is detailed in Fig. 1.

### Procedure

Participants were included between 30 October 2019 and 9 June 2020. Participants were recruited using advertisements in national newspapers, social media and at hospital emergency clinics. Applicants referred themselves to the study website (www.traumastudien.se) that was set up solely for the purpose of this trial. Applicants were initially introduced to information about the study and relevant data protection legislation and were provided with the principal investigator's contact details in case they wanted to enquire further about participation. A user ID and password were created for each participant during the registration process, which was used to access the assessment questionnaires and the intervention together with a two-factor authentication procedure.

Participants signed a digital informed consent form before completing the online screening forms. The online screening form consisted of general demographic questions, questions about the index trauma, trauma history, a checklist of inclusion and exclusion criteria, and several self-report questionnaires: PCL-5 (Blevins, Weathers, Davis, Witte, & Domino, 2015), Montgomery Åsberg Depression Rating Scale – Self-rated version (MADRS-S; Svanborg & Asberg, 1994), Alcohol Use Disorders Identification Test (AUDIT; Saunders, Aasland, Babor, de la Fuente, & Grant, 1993) and the Drug Use Disorders Identification Test (Berman, Bergman, Palmstierna, & Schlyter, 2005). Applicants who did not meet eligibility criteria were notified by phone and, if needed, given advice on how to access regular care. Potentially eligible applicants were scheduled for a phone interview, typically conducted on one of the following weekdays after registration, and assessed for inclusion and exclusion criteria in detail by a clinical psychologist or a trained student in their final semester of a 5-year clinical psychology programme under supervision. During the phone interview, the assessor administered the MINI 7.0.0 (Sheehan et al., 1998), a brief diagnostic interview designed to assess for 17 DSM/ICD diagnoses. The students had received extensive training in the use of MINI and structured diagnostic interviews overall (20 h and at least 4 months full-time internship). To further ensure the reliability of the participant sample, the first and last author reviewed each case and in some cases called participants for a second opinion or for clarifications. Eligible participants were instructed to log in to the study website and complete the baseline assessment. After completing the baseline measures, the participant was formally included in the trial and randomised to either of the conditions. The intervention typically started on the first weekday following the randomisation. Participants were assessed with the PCL-5 and a structured self-report questionnaire used to capture adverse events associated with the intervention at baseline, week 1, week 2 and week 3 (primary endpoint) and week 4, week 5, week 6 and week 7 (secondary endpoint). The weekly measures were scheduled to be filled out on the last weekday of each week. The secondary outcome measures were filled out at baseline, week 3 (post-intervention) and week 7 (1-month follow-up). In addition, the participants could report potential adverse events during the study period through the message function on the Internet platform during the intervention or by contacting the study personnel by phone. At the 1-month follow-up, participants were asked to report any changes in their psychotropic medication or psychological treatment. If changes to psychotropic medication were made or participants commenced another form of CBT-T treatment, we considered that a deviation from the study protocol. All primary and secondary measures were administered at the 6-month follow-up.

### Randomisation

Participants were randomised without constraints on a 1:1 ratio to the intervention or waiting list control condition. The randomisation list was created by an independent party (www.random.org) using a true random algorithm and the assessors were blinded to group allocation.

### Outcomes

The primary outcome measure was the PCL-5 adapted to be used within one month of the traumatic event. The original PCL-5 assesses the 20 PTSD symptoms as outlined in the DSM-5 during the past month on a four-point scale creating a possible total symptom severity score of 0–80 with higher scores indicating greater severity (Blevins et al., 2015). As the index trauma event had occurred very recently for many participants in this trial, we defined the recall period to ‘since the incident’ at screening and baseline assessments. At weeks 1–7, the recall period was set to ‘during the last 7 days’. A cut-off of 29 has been found to be indicative of probable PTSD in the non-altered Swedish version of PCL-5 (Bondjers, 2020).

Secondary measures included the MADRS-S, which assesses symptoms of depression (Svanborg & Åsberg, 2001), and EuroQol-5 Dimension (EQ-5D; Rabin & de Charro, 2001) as a measure of quality of life. A structured adverse events questionnaire that has been used in a previous trial with similar results to face-to-face interviews (Andersson et al., 2015) was used to capture the frequency and nature of possible adverse events. The participants were asked to report and rate the short- and long-term discomfort of any adverse event on a scale from 0 (‘did not affect me at all’) to 3 (‘affected me very negatively’).

### Intervention

CIPE is a 3-week therapist-guided online intervention that was provided via a secure study website. The intervention is based on the prolonged exposure (PE) protocol (Foa, Hembree, Rauch, & Rothbaum, 2019). PE is an exposure-based form of CBT-T with considerable empirical support for the treatment of PTSD (Cusack et al., 2016). The protocol typically consists of up to 15 individual weekly 90 min sessions (Foa et al., 2019). In CIPE, the PE protocol was adapted to allow guided Internet delivery, to fit as an early intervention, and the intervention length was shortened.

CIPE comprises of four modules that participants sequentially gain access to after completing homework exercises. The modules are text-based and the content is also available as audio files. Participants were informed that the intervention required approximately 6 h per week of their time during the 3-week intervention period. In order to make full use of the short intervention period, the participants were encouraged to have daily contact with their psychologist. To ensure a shared understanding of the intervention components, each module ends with a short quiz of the module content. In the quizzes, the participants are asked to summarise the central intervention component of the specific module. The therapists did not give participants access to the following module until they have been assessed as adhering to the module.

Participants could expect to receive a response from their psychologist through an email system within the intervention platform within 24 h on weekdays. The therapists were instructed to guide the participants through the treatment by answering questions, providing support and encouragement on the progress made, and to provide individually tailored feedback on completed assignments, on participants' progress, and/or to troubleshoot eventual difficulties. If a participant did not log in to the platform for 3 days or was late in submitting the homework exercises, the psychologist either sent out reminders via the online platform in the form of short text messages or email, or called the participant by telephone.

### The therapists

Most participants (95%) were supported by one of four clinical psychologists (M.B., K.A., J.S. and K.O.L.). The remaining 5% were supported by one of five trained students in their final semester of a 5-year clinical psychology programme under the supervision of M.B. All therapists received a 3 h training session in the CIPE protocol, which was developed by M.B. and E.A. M.B. has been extensively trained by the developer of PE, Professor Edna Foa, and is a certified supervisor and trainer in PE. In addition, M.B. monitored all participants and provided supervision to the therapists on demand. The students were supervised by M.B. on a weekly basis.

### The intervention content

As in the original PE protocol (Foa et al., 2019), CIPE includes the treatment components psychoeducation, *in vivo* exposure and imaginal exposure. Controlled breathing was taught as a skill to reduce general stress, as in the original PE protocol.

The first module is an introduction to the intervention and includes psychoeducation about common reactions after exposure to psychological trauma. The module also introduces controlled breathing as a way for the participant to manage general stress. Participants are encouraged to practice controlled breathing three times a day. The purpose of this module is to normalise and validate the participants' reactions and instil hope of recovery from the symptoms of post-traumatic stress.

The second module focuses on imaginal exposure: that is, to revisit and recount the memory of the traumatic event. First, a rationale is provided for the presumed role of imaginal exposure in reducing symptoms of post-traumatic stress and instructions on how to conduct imaginal exposure. Case examples are used to illustrate how to revisit the traumatic memory and were constructed to include common difficulties that may arise during imaginal exposure, such as over-engagement, under-engagement and experiencing dissociative reactions (Foa et al., 2019) together with suggestions on how to overcome them. The participants are encouraged to revisit the traumatic memory daily for 20–30 min each time, either by recording a verbal recount or writing down their trauma narrative using either pen and paper or digitally. The recommended time spent on imaginal exposure each day was motivated by studies suggesting no differences in treatment effects when using 30 min (van Minnen & Foa, 2006), 20 min (Nacasch et al., 2015) or 10 min of imaginal exposure (Bryant et al., 2019) compared to the stipulated 45 min in the original PE protocol (Foa et al., 2019). The participants are instructed to set aside an additional 15 min after each imaginal exposure for cognitive processing and to reflect upon any corrective learning experiences that could arise from the imaginal exposure. Through the use of digital worksheets, the participants are assisted to observe changes in perceived emotional intensity during the exposure exercise and to notice changes in negative beliefs about themselves, others or the world. The participants registered each exposure exercise in a pre-defined worksheet in the online platform which helped the psychologist to keep track of their progress. Each worksheet could be duplicated and filled out an infinite number of times by the participant.

Module three expands the imaginal exposure exercise and participants are encouraged to approach the most distressing sequences of their traumatic memory (i.e. hot spots). Module three also targets behavioural avoidance and includes a rationale for and instructions on how to approach situations that are perceived as dangerous or triggering trauma-related distress but are objectively safe. Participants are asked to compose a list of avoided situations and rank them in order from least to most distressing. The participants are then encouraged to gradually confront these situations, starting with those that evoke moderate distress. The online therapist assisted in planning the *in vivo* exposures through email contact and by reading the worksheets, and took extensive measures to ensure that the participant did not approach objectively dangerous situations. Module three also contains case examples to illustrate common challenges when conducting *in vivo* exercises (e.g. engaging in safety behaviours) and how to overcome these.

Module four includes a summary of the intervention and a rationale for how to continue to use the strategies learned from the intervention. The participant is asked to summarise their own progress and to make an individual plan for relapse prevention. The participants were asked to use a digital worksheet to review their progress and what they had learned from the intervention, to reflect upon how these changes were accomplished, and how to deal with any temporary increases in symptoms of post-traumatic stress in the future. In the last part of the module, the participants were reminded of the upcoming follow-up assessments and were asked to summarise their experience of the intervention. The participants continued to have access to the intervention for one year after completing the intervention but without therapist support.

### Control group

Participants randomised to the waiting list condition were informed of the weekly assessments and that they would receive delayed intervention after 7 weeks. Participants were provided with a telephone number to a study psychologist in case of acute worsening of symptoms or suicidal ideation. In case of worsening, the study personnel helped the participants to contact their regular health care providers.

### Power calculation

Based on our pilot trial (Bragesjö et al., 2021a) we expected a moderate to large effect size at week 3, our primary endpoint, on the PCL-5. Given 95% power, 10% data attrition rate and an *α* level of 0.05, we calculated that we would need a total of 100 participants to find a statistically significant moderate between-group effect of *d* = 0.6 at week 3. In order to increase power further, the primary outcome was administered weekly during the intervention.

### Statistical analyses

Primary analyses were conducted in STATA 16.1 according to the intention-to-treat principle. The efficacy of the intervention was evaluated using a mixed-effects regression framework with maximum likelihood estimation. The advantage of this type of analysis is that all available data are used to estimate the intervention effects based on the distribution of observed data points, instead of relying on only those who have complete data (Lane, 2008; Mallinckrodt, Clark, & David, 2001). The model included fixed effects of group (CIPE *v.* waiting list), time (baseline and week 1–7), group × time interaction and random intercepts. The m_effectsize command in Stata [publicly available using the STATA command ‘net install m_effectsize, from (http://www.imm.ki.se/biostatistics/stata) replace’] was used to estimate the magnitude of the treatment effects. This command estimates effect sizes (Cohen's *d*) by dividing the estimated change score in the mixed-effects regression analysis (the estimated group × time interaction based on data from all weekly measures) by the pooled standard deviation at baseline. In order to construct a 95% confidence interval of the estimated effect size, 1000 bootstrap replications were used. Effect sizes were categorized according to Cohen's recommendations, with small, moderate and large effect sizes corresponding to *d* = 0.20, 0.50 and 0.80, respectively (Cohen, 1992). We classified participants as responders if they had a reduction on the PCL-5 total score ⩾10 points (Weathers et al., 2013). Logistic regression was used to estimate between-group differences in responder rates.

## Results

Table 1 presents baseline characteristics of the recruited participants (*N* = 102). The majority were women and their mean age was 41. About half of the sample had a college or university degree. Approximately one-third were on sick leave. The most common index traumas were exposure to interpersonal violence (28%), death (25%), rape (16%) and motor vehicle accidents (11%). The larger part of participants (75%) had directly experienced the index traumatic event. About half of the participants reported that they had sought medical attention after exposure to their index trauma and approximately one-fifth reported having been admitted to a hospital. Participants were included, on average, slightly more than 1 month after the event. More than two-thirds of the sample fulfilled the criteria for a current psychiatric diagnosis. As seen in the trial flowchart (Fig. 1), data attrition was fairly low at both the primary (7.8%) and secondary (10.8%) endpoints. One participant in each group dropped out of the study and did not want to be contacted any more. On average, each therapist spent less than 1 h (*M* = 46 min; s.d. = 48) on each participant during the intervention period of 3 weeks. As for adherence, 33 participants (65%) in the intervention group completed at least three modules.

**Table 1.** Sociodemographic and clinical characteristics of participants at baseline

Baseline characteristic		CIPE^(*N* = 51)^	WL^(*N* = 51)^
Gender, *n* (*%*)	Women	45 (88%)	39 (76%)
Age	Mean (s.d.)	44.7 (17)	36.7 (13.4)
	Range	18–82	18–75
Highest education, *n* (*%*)	College/university	30 (57%)	25 (47%)
Occupational status, *n* (*%*)	Working full time	15 (29%)	20 (39%)
	On sick leave	17 (33%)	15 (29%)
Psychiatric diagnoses according to the MINI, *n* (*%*)	PTSD	22 (43%)	23 (45%)
	Current depressive episode	21 (41%)	31 (60%)
	Anxiety disorder or OCD	14 (27%)	14 (27%)
	Any	35 (68%)	38 (74%)
Prior exposure to trauma, *n* (%)	In childhood	3 (6%)	8 (16%)
	In adulthood	16 (31%)	14 (27%)
	Both child and adult trauma	18 (35%)	17 (33%)
	None	14 (27%)	12 (24%)
Type of trauma, *n* (%)	Rape/interpersonal violence	27 (53%)	28 (55%)
	Non-intentional	24 (47%)	23 (45%)
Days since exposure to index trauma, mean (s.d.)	Mean (s.d.)	36.3 (19)	35.4 (17.11)
	Range	6–65	4–62

CIPE, Condensed Internet-delivered Prolonged Exposure; WL; waiting list; MINI, Mini International Neuropsychiatric Interview; OCD, obsessive-compulsive disorder; PTSD, post-traumatic stress disorder, WL; waiting list.

### Efficacy

**Fig. 2.**
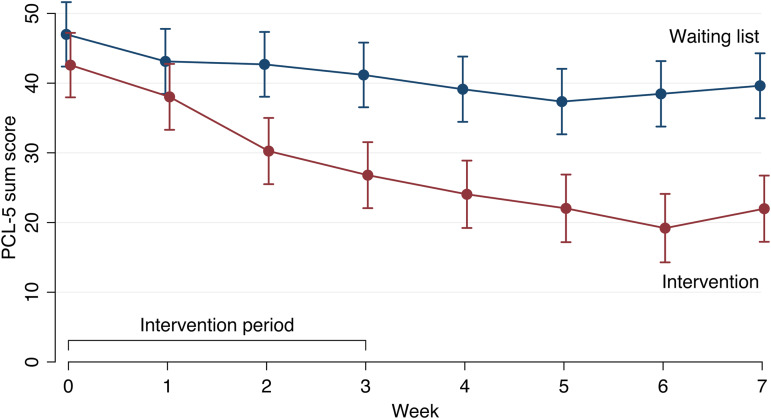
Change in symptoms of post-traumatic stress during the controlled study period.

Table 2 displays means, standard deviation and effect sizes for the continuous outcome measures at the assessment points during the trial and provide *p* values for the interaction effect estimates and effect sizes (*d*) with 95% bootstrapped confidence intervals. There was a larger reduction in symptoms of post-traumatic stress in the CIPE group than in the waiting list at both the primary and secondary endpoint. The between-group effect size was moderate at week 3 and large at week 7. The mean improvement in PCL-5 sum score was 16.29 points in the CIPE group compared to 5.56 points in the waiting list group at week 3. The difference was even larger at week 7, at which timepoint the mean improvement was 20.36 points in the CIPE group and 6.86 points in the waiting list group (Fig. 2). Results were sustained in the intervention group from week 7 to the 6-month follow-up (*β* = 1.35, *z* = 0.53, *p* = 0.53). Detailed information about the distribution is depicted in eFig. 1 and eFig. 2.

**Table 2.** Observed values and change in symptoms from baseline to week 7 on the outcome measures

	CIPE (*n* = 51)		Waiting list (*n* = 51)		Group × time interaction effect			Effect size
Variable	*M*	(s.d.)	*M*	(s.d.)	*β*-value	*Z*-value	*p* value	Bootstrapped d (95% CI)
PCL-5								
Baseline	42.59	(14.25)	47.00	(14.02)				
Week 1	38.09	(16.75)	42.51	(13.43)				
Week 2	30.74	(17.26)	42.94	(15.82)				
Week 3	26.30	(19.31)	41.42	(16.50)	14.50	3.83	<0.0001	0.71 (0.33–1.05)
Week 4	23.63	(18.93)	39.60	(16.92)				
Week 5	20.81	(18.92)	37.69	(17.52)				
Week 6	18.02	(17.17)	38.64	(17.67)				
Week 7	22.23	(20.33)	40.14	(18.91)	17.67	4.41	<0.0001	0.83 (0.46–1.19)
6-month follow-up	18.59	(17.80)	26.48	(17.73)				
MADRS-S								
Baseline	22.17	(10.10)	24.62	(8.82)				
Week 3	17.02	(11.84)	22.70	(9.25)	2.86	1.95	0.052	0.30 (0.02–0.60)
Week 7	14.93	(11.35)	21.72	(10.61)	4.04	2.39	0.02	0.40 (0.08–0.72)
6-month follow-up	12.34	(10.35)	15.43	(9.80)				
EQ-5D								
Baseline	0.49	(0.34)	0.51	(0.34)				
Week 3	0.67	(0.33)	0.56	(0.33)	0.06	1.90	0.06	0.35 (0.05–0.65)
Week 7	0.69	(0.30)	0.55	(0.31)	0.07	2.04	0.04	0.40 (0.07–0.75)
6-month follow-up	0.75	(0.27)	0.66	(0.29)				

CIPE, Condensed Internet-delivered Prolonged Exposure; WL; waiting list; PCL-5, PTSD Checklist for DSM-5; MADRS-S, Montgomery Åsberg Depression Rating Scale – Self-rated version; EQ-5D, EuroQol-5 Dimension.

In the analysis of responder rates on observed values, 25 participants (60%) were classified as responders in the CIPE group at week 3, compared to 11 (23%) in the control group (RR = 2.54, χ^2^ = 12.01, *p* = 0.0005). At week 7, 38 (75%) participants were classified as responders in the CIPE group compared to 20 (40%) in the waiting list control group (RR = 1.9, χ^2^ = 12.95, *p* = 0.003).

For the secondary outcome measures, the same trend in reductions in symptoms of depression and quality of life was seen at week 7 but not at week 3. The intervention group had a greater decrease in symptoms of depression than the waiting list at week 7. In addition, CIPE conferred a significantly larger increase in the quality of life at week 7. The observed between-group effect sizes were small, see Table 2.

### Changes in PTSD symptom clusters

Post hoc analyses were conducted on the subscales on the PCL-5 (eFig. 3). The mixed-effects model showed a significant interaction effect on all of the PCL-5 subscales. The between-group effect sizes were large for the avoidance subscale at week 3 and week 7. For the intrusion and cognitions and mood subscales, the between-group effect sizes were moderate at week 3 and large at week 7. The between-group effect size for the hyperarousal subscale was small at week 3 and moderate at week 7.

### Protocol deviations and adverse events

Three protocol deviations were reported during the controlled study period of 7 weeks, all in the waiting list group condition. Two participants reported changes in their prescribed type of antidepressant and one reported discontinuation of taking antidepressants. The efficacy analyses were run again while excluding these participants and the results remained the same (data not shown) as in the primary analysis.

In the CIPE group, 16 participants (31%) reported a total of 63 adverse events (Table 3). The reported adverse events were rated as mild to moderate. The mean short- and long-term discomfort associated with each adverse event on the three-point scale was 2.3 and 1.0, respectively. In the waiting list, 11 participants (21%) reported a total of 35 adverse events. The mean short- and long-term discomfort associated with each adverse event was 2.1 and 2.0, respectively.

**Table 3.** Frequency and types of adverse events during the controlled study period

Adverse event	CIPE (*n* = 16)	WL (*n* = 11)
Increase in number of intrusive memories of the index event	3 (5.8%)	1 (1.9%)
Increase in number of intrusive memories from previous traumatic events	2 (3.8%)	–
Initial symptom exacerbation	1 (1.9%)	–
Increase in depressive symptoms	5 (9.6%)	1 (1.9%)
Increase in anxiety	4 (7.7%)	8 (15.4%)
Sleep problems	1 (1.9%)	–
Panic attacks	2 (3.8%)	1 (1.9%)
Increase in stress	3 (5.8%)	–
Anger/irritability	6 (11.5%)	–
Severe distress during exposure	5 (9.6%)	–
Tiredness	1 (1.9%)	–
Memory impairment	1 (1.9%)	–
Migraine	1 (1.9%)	–
Increase in impulsivity	2 (3.8%)	–
Increased pain	2 (3.8%)	–

Note that in the 27 participants that reported adverse events, the average number of reported events were 4 in the CIPE group and 3 in the waiting list group. CIPE, Condensed Internet-delivered Prolonged Exposure; WL, waiting list.

## Discussion

The current study was a randomised trial with 102 participants recently exposed to trauma and experiencing symptoms of post-traumatic stress, comparing the digital intervention CIPE against a waiting list. The results indicated that the CIPE group had significantly larger reductions in symptoms of post-traumatic stress than the waiting list. Moderate-to-large effect sizes were observed at post-assessment and 1 month after the intervention. The intervention also conferred some benefit with regard to symptoms of depression and quality of life as compared to the control group. The results concur with the data from our pilot trial (Bragesjö et al., 2021*a*) and further extend the conclusions that CIPE is an effective intervention as this trial used a larger sample and had a longer controlled follow-up time of 7 weeks.

The current trial is the first to evaluate a digital, therapist-guided exposure-based intervention provided in the early phase after trauma in a larger RCT. The effect sizes found in this trial are comparable to or larger than those found in studies that have evaluated CBT-T as an early intervention when delivered face-to-face (Bryant et al., 2008; Maples-Keller et al., 2020; Rothbaum et al., 2012). Thus, the results found in this study provide further enthusiasm for intervening early to reduce post-traumatic stress as it corroborates previous findings showing positive effects of exposure-based interventions in the early aftermath of trauma.

The results in this trial clearly show that it is possible to reach and deliver interventions to trauma-afflicted individuals remotely using a digital platform. CIPE is a flexible intervention as it does not require any scheduled appointments or participants to commute to a clinic. Together with the anonymity of the digital format for trauma victims struggling with shame (Kantor, Knefel, & Lueger-Schuster, 2017), this might lower the threshold for trauma survivors to seek out and receive help. The findings from this trial are further encouraging as CIPE showed clinically meaningful effects while needing only a small amount of therapist resources (46 min per participant). CIPE has the potential to increase the supply of evidence-based treatments to a larger population of trauma-exposed individuals with little or no increase in the existing limited therapist resources. This is of particular relevance in large-scale crises that challenge and put pressure on existing health-care services, ranging from natural disasters, terror attacks to prolonged events such as the ongoing Covid-19 pandemic (Holmes et al., 2020; Javakhishvili et al., 2020).

There are concerns among clinicians that exposure-based interventions may not be tolerable for many trauma-afflicted individuals (Deacon & Farrell, 2013; Jonas et al., 2013). In contrast, although participants in CIPE reported a greater number of adverse events, these adverse events were of mild to moderate severity in both the CIPE and waiting list, and none of the participants reported any serious adverse events. Other indicators of tolerability were the low frequency of dropouts and a high degree of adherence. A recent review found indications of elevated dropout rates in patients undergoing Internet-based CBT for PTSD (Simon et al., 2019) and a trial that evaluated an automated digital early intervention that shares components with CIPE such as psychoeducation and *in vivo* exposure reported a low usage of the intervention (Mouthaan et al., 2013). In this current trial, however, the dropout rate was low and the adherence was high. In a qualitative study by our research group, participants who received CIPE described the therapists' support as essential in motivating them to do anxiety-provoking exercises such as imaginal exposure (Bragesjö et al., 2021*b*). It is thus reasonable to assume that the frequent yet brief therapist contact in CIPE is important to increase compliance and minimize dropout. From a clinical perspective, CIPE may also have some specific benefits. For example, the firm structure may reduce the risk of therapist drift and ensure consistent provision of the content.

The main strengths of the current trial study were the randomised design, the large sample size, the use of repeated measures as well as the controlled follow-up time of 1 month after treatment completion. The trial also acknowledges limitations and provides important research questions for future research. One important methodological limitation of this trial was that the outcome measures were self-report questionnaires. The groups may have differed in implicit expectation on how to report their symptoms, which may have introduced a bias towards greater effects. Future trials would benefit from using clinician-administered assessments using blinded assessors. Second, the negative effects on natural recovery seen in a small group of trauma victims provided with critical incident stress debriefing immediately post-trauma were not found until 18 months later (Rose, Bisson, Churchill, & Wessely, 2002). It is therefore also important to further investigate the long-term effects of CIPE. A third limitation with the current trial is the use of a waiting list, which may have generated inflated effect sizes (Gold et al., 2017). On the other hand, this specific trial tested an early intervention for individuals who experienced a traumatic event and previous research has shown that a substantial portion of people exposed to trauma naturally recover with the passage of time (Koenen et al., 2017). A further limitation with the current study is the self-selected sample, in that it may introduce uncertainty as to whether the findings herein are generalisable to a wider population of trauma-exposed individuals that healthcare services would want to target with this type of intervention. However, the sample in the current trial shared the same characteristics as generally seen in PTSD patients. The trauma types were similar to those reported in The World Mental Health Surveys as the leading precursors of PTSD (Benjet et al., 2016). The high rates of psychiatric comorbidity in the sample were also comparable to what is typically found in patients with PTSD. Evaluation of the CIPE intervention is currently underway in a primary care context and we plan to compare and benchmark the effects against the results found in this trial during 2021. Another research topic for the future would be to test the hypothesized mechanism of change. Studies have suggested that negative cognitions about oneself and the world are a central mediator of change in exposure-based treatments for PTSD (e.g. Kumpula et al., 2017; Zalta et al., 2014) and a subsequent step would be to investigate whether this is also the case in CIPE. Also, this study did not assess intervention usage after the acute phase of treatment and future studies should investigate this further.

Notwithstanding its limitations, this trial provides evidence that CIPE is efficacious in reducing short- and long-term symptoms of post-traumatic stress compared to a waiting list condition. The trial is the largest to date showing that it is safe and feasible to deliver exposure-based interventions in the early post-trauma phase with remotely-delivered therapist support. CIPE holds the potential to increase the outreach to the millions of people exposed to psychologically traumatic events each year.

## Supplementary material

For supplementary material accompanying this paper visit https://doi.org/10.1017/S0033291721003706. click here to view supplementary material

## References

[ref1] American Psychiatric Association (2013). Diagnostic and statistical manual of mental disorders (5th ed.). Arlington, VA: American Psychiatric Publishing. doi:10.1176/appi.books.9780890425596

[ref2] Andersson, E., Hedman, E., Enander, J., Radu Djurfeldt, D., Ljotsson, B., Cervenka, S., … Ruck, C. (2015). D-cycloserine vs placebo as adjunct to cognitive behavioral therapy for obsessive-compulsive disorder and interaction with antidepressants: A randomized clinical trial. JAMA Psychiatry, 72(7), 659–667. doi: 10.1001/jamapsychiatry.2015.0546 25970252

[ref3] Benjet, C., Bromet, E., Karam, E. G., Kessler, R. C., McLaughlin, K. A., Ruscio, A., … Koenen, K. C. (2016). The epidemiology of traumatic event exposure worldwide: Results from the World Mental Health Survey Consortium. Psychological Medicine, 46(2), 327–343. doi: 10.1017/s0033291715001981 26511595 PMC4869975

[ref4] Berman, A. H., Bergman, H., Palmstierna, T., & Schlyter, F. (2005). Evaluation of the Drug Use Disorders Identification Test (DUDIT) in criminal justice and detoxification settings and in a Swedish population sample. European Addiction Research, 11(1), 22–31. doi: 10.1159/000081413 15608468

[ref5] Blevins, C. A., Weathers, F. W., Davis, M. T., Witte, T. K., & Domino, J. L. (2015). The posttraumatic stress disorder checklist for DSM-5 (PCL-5): Development and initial psychometric evaluation. Journal of Traumatic Stress, 28(6), 489–498. doi: 10.1002/jts.22059 26606250

[ref6] Bondjers, K. (2020). Post-traumatic stress disorder – Assessment of current diagnostic definitions (PhD dissertation, Acta Universitatis Upsaliensis). Retrieved from http://urn.kb.se/resolve?urn=urn:nbn:se:uu:diva-403118.

[ref7] Bragesjö, M., Arnberg, F. K., Jelbring, A., Nolkrantz, J., Särnholm, J., Olofsdotter Lauri, K., … Andersson, E. (2021b). Demanding and effective: Participants’ experiences of internet-delivered prolonged exposure provided within two months after exposure to trauma. European Journal of Psychotraumatology, 12(1), 1885193. doi: 10.1080/20008198.2021.1885193 33968320 PMC8075080

[ref8] Bragesjö, M., Arnberg, F. K., Särnholm, J., Olofsdotter Lauri, K., & Andersson, E. (2021a). Condensed internet-delivered prolonged exposure provided soon after trauma: A randomised pilot trial. Internet Interventions, 23, 100358. doi: 10.1016/j.invent.2020.100358 33384946 PMC7771112

[ref9] Bragesjö, M., Larsson, K., Nordlund, L., Anderbro, T., Andersson, E., & Möller, A. (2020). Early psychological intervention after rape: A feasibility study. Frontiers in Psychology, 11, 1595–1602. doi:10.3389/fpsyg.2020.01595. 32733345 PMC7360814

[ref10] Bryant, R. A. (2003). Early predictors of posttraumatic stress disorder. Biological Psychiatry, 53(9), 789–795. doi: 10.1016/s0006-3223(02)01895-4 12725971

[ref11] Bryant, R. A., Kenny, L., Rawson, N., Cahill, C., Joscelyne, A., Garber, B., … Nickerson, A. (2019). Efficacy of exposure-based cognitive behaviour therapy for post-traumatic stress disorder in emergency service personnel: A randomised clinical trial. Psychological Medicine, 49(9), 1565–1573. doi: 10.1017/S0033291718002234 30149825

[ref12] Bryant, R. A., Mastrodomenico, J., Felmingham, K. L., Hopwood, S., Kenny, L., Kandris, E., … Creamer, M. (2008). Treatment of acute stress disorder: A randomized controlled trial. Archives of General Psychiatry, 65(6), 659–667. doi: 10.1001/archpsyc.65.6.659 18519824

[ref13] Cohen, J. (1992). A power primer. Psychological Bulletin, 112(1), 155–159. doi: 10.1037//0033-2909.112.1.155 19565683

[ref14] Cusack, K., Jonas, D. E., Forneris, C. A., Wines, C., Sonis, J., Middleton, J. C., … Gaynes, B. N. (2016). Psychological treatments for adults with posttraumatic stress disorder: A systematic review and meta-analysis. Clinical Psychological Review, 43, 128–141. doi: 10.1016/j.cpr.2015.10.003 26574151

[ref15] Deacon, B. J., & Farrell, N. R. (2013). Therapist barriers to the dissemination of exposure therapy. In E. A. Storch & D. McKay (Eds.), Handbook of treating variants and complications in anxiety disorders (pp. 363–373). New York: Springer Science + Business Media.

[ref16] Foa, E., Hembree, E. A., Rauch, S., & Rothbaum, B. O. (2019). Prolonged exposure therapy for PTSD: Emotional processing of traumatic experiences - therapist guide. New York: Oxford University Press.

[ref17] Galea, S., Ahern, J., Resnick, H., Kilpatrick, D., Bucuvalas, M., Gold, J., & Vlahov, D. (2002). Psychological sequelae of the September 11 terrorist attacks in New York City. New England Journal of Medicine, 346(13), 982–987. doi: 10.1056/NEJMsa013404 11919308

[ref18] Gold, S. M., Enck, P., Hasselmann, H., Friede, T., Hegerl, U., Mohr, D. C., & Otte, C. (2017). Control conditions for randomised trials of behavioural interventions in psychiatry: A decision framework. The Lancet Psychiatry, 4(9), 725–732. doi: 10.1016/s2215-0366(17)30153-0 28396067

[ref19] Holmes, E. A., O'Connor, R. C., Perry, V. H., Tracey, I., Wessely, S., Arseneault, L., … Everall, I. (2020). Multidisciplinary research priorities for the COVID-19 pandemic: A call for action for mental health science. The Lancet Psychiatry, 7(6), 547–560. doi:10.1016/S2215-0366(20)30168-1 32304649 PMC7159850

[ref20] Javakhishvili, J. D., Ardino, V., Bragesjö, M., Kazlauskas, E., Olff, M., & Schäfer, I. (2020). Trauma-informed responses in addressing public mental health consequences of the COVID-19 pandemic: Position paper of the European Society for Traumatic Stress Studies (ESTSS). European Journal of Psychotraumatology, 11(1), 1780782. doi: 10.1080/20008198.2020.1780782 33029320 PMC7473312

[ref21] Jonas, D. E., Cusack, K., Forneris, C. A., Wilkins, T. M., Sonis, J., Middleton, J. C., … Gaynes, B. N. (2013). Psychological and pharmacological treatments for adults with posttraumatic stress disorder (PTSD) (Report No.: 13-EHC011-EF). Rockville: Agency for Healthcare Research and Quality (US). 23658937

[ref22] Kantor, V., Knefel, M., & Lueger-Schuster, B. (2017). Perceived barriers and facilitators of mental health service utilization in adult trauma survivors: A systematic review. Clinical Psychology Review, 52, 52–68. doi: 10.1016/j.cpr.2016.12.001 28013081

[ref23] Kessler, R. C., Sonnega, A., Bromet, E., Hughes, M., & Nelson, C. B. (1995). Posttraumatic stress disorder in the national comorbidity survey. Archives of General Psychiatry, 52(12), 1048–1060. doi: 10.1001/archpsyc.1995.03950240066012 7492257

[ref24] Koenen, K. C., Ratanatharathorn, A., Ng, L., McLaughlin, K. A., Bromet, E. J., Stein, D. J., … Kessler, R. C. (2017). Posttraumatic stress disorder in the World Mental Health Surveys. Psychological Medicine, 47(13), 2260–2274. doi: 10.1017/S0033291717000708 28385165 PMC6034513

[ref25] Kumpula, M. J., Pentel, K. Z., Foa, E. B., LeBlanc, N. J., Bui, E., McSweeney, L. B., … Rauch, S. A. (2017). Temporal sequencing of change in posttraumatic cognitions and PTSD symptom reduction during prolonged exposure therapy. Behavior Therapy, 48(2), 156–165. doi:10.1016/j.beth.2016.02.008 28270327

[ref26] Lane, P. (2008). Handling drop-out in longitudinal clinical trials: A comparison of the LOCF and MMRM approaches. Pharmaceutical Statistics, 7(2), 93–106. doi: 10.1002/pst.267 17351897

[ref27] Mallinckrodt, C. H., Clark, W. S., & David, S. R. (2001). Accounting for dropout bias using mixed-effects models. Journal of Biopharmaceutical Statistics, 11(1–2), 9–21. doi: 10.1081/BIP-100104194 11459446

[ref28] Maples-Keller, J. L., Post, L. M., Price, M., Goodnight, J. M., Burton, M. S., Yasinski, C. W., … Rothbaum, B. O. (2020). Investigation of optimal dose of early intervention to prevent posttraumatic stress disorder: A multiarm randomized trial of one and three sessions of modified prolonged exposure. Depression and Anxiety, 37(5), 429–437. doi: 10.1002/da.23015 32248637 PMC7347250

[ref29] McFarlane, A. C., Atchison, M., Rafalowicz, E., & Papay, P. (1994). Physical symptoms in post-traumatic stress disorder. Journal of Psychosomatic Research, 38(7), 715–726. doi: 10.1016/0022-3999(94)90024-8 7877126

[ref30] Mouthaan, J., Sijbrandij, M., de Vries, G.-J., Reitsma, J. B., van de Schoot, R., Goslings, J. C., … Olff, M. (2013). Internet-based early intervention to prevent posttraumatic stress disorder in injury patients: Randomized controlled trial. Journal of Medical Internet Research, 15(8), e165. doi: 10.2196/jmir.2460 23942480 PMC3742408

[ref31] Nacasch, N., Huppert, J. D., Su, Y. J., Kivity, Y., Dinshtein, Y., Yeh, R., & Foa, E. B. (2015). Are 60-minute prolonged exposure sessions with 20-minute imaginal exposure to traumatic memories sufficient to successfully treat PTSD? A randomized noninferiority clinical trial. Behavior Therapy, 46(3), 328–341. doi: 10.1016/j.beth.2014.12.002 25892169

[ref32] Rabin, R., & de Charro, F. (2001). EQ-5D: A measure of health status from the EuroQol group. Annals of Medicine, 33(5), 337–343. doi: 10.3109/07853890109002087 11491192

[ref33] Rose, S., Bisson, J., Churchill, R., & Wessely, S. (2002). Psychological debriefing for preventing post traumatic stress disorder (PTSD). Cochrane Database of Systematic Reviews, (2), CD000560. doi: 10.1002/14651858.CD000560 12076399

[ref34] Rothbaum, B. O., Foa, E. B., Riggs, D. S., Murdock, T., & Walsh, W. (1992). A prospective examination of post-traumatic stress disorder in rape victims. Journal of Traumatic Stress, 5(3), 455–475. doi: 10.1007/bf00977239

[ref35] Rothbaum, B. O., Kearns, M. C., Price, M., Malcoun, E., Davis, M., Ressler, K. J., … Houry, D. (2012). Early intervention may prevent the development of posttraumatic stress disorder: A randomized pilot civilian study with modified prolonged exposure. Biological Psychiatry, 72(11), 957–963. doi:10.1016/j.biopsych.2012.06.002 22766415 PMC3467345

[ref36] Saunders, J. B., Aasland, O. G., Babor, T. F., de la Fuente, J. R., & Grant, M. (1993). Development of the alcohol use disorders identification test (AUDIT): WHO collaborative project on early detection of persons with harmful alcohol consumption – II. Addiction, 88(6), 791–804. doi: 10.1111/j.1360-0443.1993.tb02093.x 8329970

[ref37] Sheehan, D. V., Lecrubier, Y., Sheehan, K. H., Amorim, P., Janavs, J., Weiller, E., … Dunbar, G. C. (1998). The Mini-International Neuropsychiatric Interview (M.I.N.I.): The development and validation of a structured diagnostic psychiatric interview for DSM-IV and ICD-10. Journal of Clinical Psychiatry, 59(Suppl 20), 22–57. 9881538

[ref38] Simon, N., McGillivray, L., Roberts, N. P., Barawi, K., Lewis, C. E., & Bisson, J. I. (2019). Acceptability of internet-based cognitive behavioural therapy (i-CBT) for post-traumatic stress disorder (PTSD): A systematic review. European Journal of Psychotraumatology, 10(1), 1646092. doi: 10.1080/20008198.2019.1646092 31497259 PMC6719262

[ref39] Simon, N., Robertson, L., Lewis, C., Roberts, N. P., Bethell, A., Dawson, S., … Bisson, J. I. (2021). Internet-based cognitive and behavioural therapies for post-traumatic stress disorder (PTSD) in adults. Cochrane Database of Systematic Reviews 5(5), CD011710. doi: 10.1002/14651858.CD011710.pub3 PMC8136365 34015141

[ref40] Song, H., Fang, F., Tomasson, G., Arnberg, F. K., Mataix-Cols, D., Fernandez de la Cruz, L., … Valdimarsdottir, U. A. (2018). Association of stress-related disorders with subsequent autoimmune disease. JAMA, 319(23), 2388–2400. doi: 10.1001/jama.2018.7028 29922828 PMC6583688

[ref41] Svanborg, P., & Asberg, M. (1994). A new self-rating scale for depression and anxiety states based on the Comprehensive Psychopathological Rating Scale. Acta Psychiatrica Scandinavica, 89(1), 21–28. doi: 10.1111/j.1600-0447.1994.tb01480.x 8140903

[ref42] Svanborg, P., & Åsberg, M. (2001). A comparison between the Beck Depression Inventory (BDI) and the self-rating version of the Montgomery Åsberg Depression Rating Scale (MADRS). Journal of Affective Disorders, 64(2–3), 203–216. doi: 10.1016/S0165-0327(00)00242-1 11313087

[ref43] van Minnen, A., & Foa, E. B. (2006). The effect of imaginal exposure length on outcome of treatment for PTSD. Journal of Traumatic Stress, 19(4), 427–438. doi: 10.1002/jts.20146 16929519

[ref44] Weathers, F. W., Litz, B. T., Keane, T. M., Palmieri, P. A., Marx, B. P., & Schnurr, P. P. (2013). The PTSD checklist for DSM-5 (PCL-5). Retrieved from https://www.ptsd.va.gov.

[ref45] Zalta, A. K., Gillihan, S. J., Fisher, A. J., Mintz, J., McLean, C. P., Yehuda, R., & Foa, E. (2014). Change in negative cognitions associated with PTSD predicts symptom reduction in prolonged exposure. Journal of Consulting and Clinical Psychology, 82(1), 171–175. doi: 10.1037/a0034735 24188512 PMC3951922

