# Condensed Internet-delivered prolonged exposure provided soon after trauma: a randomised trial – Corrigendum

**DOI:** 10.1017/S0033291723002027

**Published:** 2023-09

**Authors:** Maria Bragesjö, Filip K. Arnberg, Klara Olofsdotter Lauri, Kristina Aspvall, Josefin Särnholm, Erik Andersson

**Affiliations:** 1 Department of Clinical Neuroscience, Division of Psychology, Nobels väg 9, Karolinska Institutet, 171 77 Stockholm, Sweden; 2 Department of Neuroscience, Psychiatry, National Centre for Disaster Psychiatry, 751 24 Uppsala, Sweden; 3 Stress Research Institute, Stockholm University, 106 91 Stockholm, Sweden; 4 Department of Clinical Neuroscience, Centre for Psychiatry Research, Karolinska Institutet, 171 77 Stockholm, Sweden; 5 Stockholm Health Care Services, Region Stockholm, Stockholm, Sweden

When this article was published in Psychological Medicine, it contained an error in the following paragraph of text:

*In the analysis of responder rates on observed values, 25 participants* (*60%*) *were classified as responders in the CIPE group at week 3, compared to 11* (*23%*) *in the control group* (*RR = 2.54,* χ*2 = 12.01, p = 0.0005*). *At week 7, 38* (*75%*) *participants were classified as responders in the CIPE group compared to 20* (*40%*) *in the waiting list control group* (*RR = 1.9,* χ*2 = 12.95, p = 0.003*).

The correct text is as follows:

*In the analysis of responder rates on observed values, 26 participants* (*59%*) *were classified as responders in the CIPE group at week 3, compared to 12* (*24%*) *in the control group* (*RR = 2.46,* χ*2 = 11.97, p = 0.0005*). *At week 7, 31* (*72%*) *participants were classified as responders in the CIPE group compared to 18* (*38%*) *in the waiting list control group* (*RR = 1.92,* χ*2 = 10.92, p = 0.001*).

The authors apologise for this error.
