# Supplementary material for: Condensed Internet-delivered prolonged exposure provided soon after trauma: a randomised trial
Source: Psychol Med. 2021 Sep 14;53(5):1989–98. doi: 10.1017/S0033291721003706 (PMC10106292; doi:10.1017/S0033291721003706)
Supplement: Supplementary file 1 [file S0033291721003706sup001.docx]

**Supplement**

**Efficacy**

**Change from baseline to week 3**

**
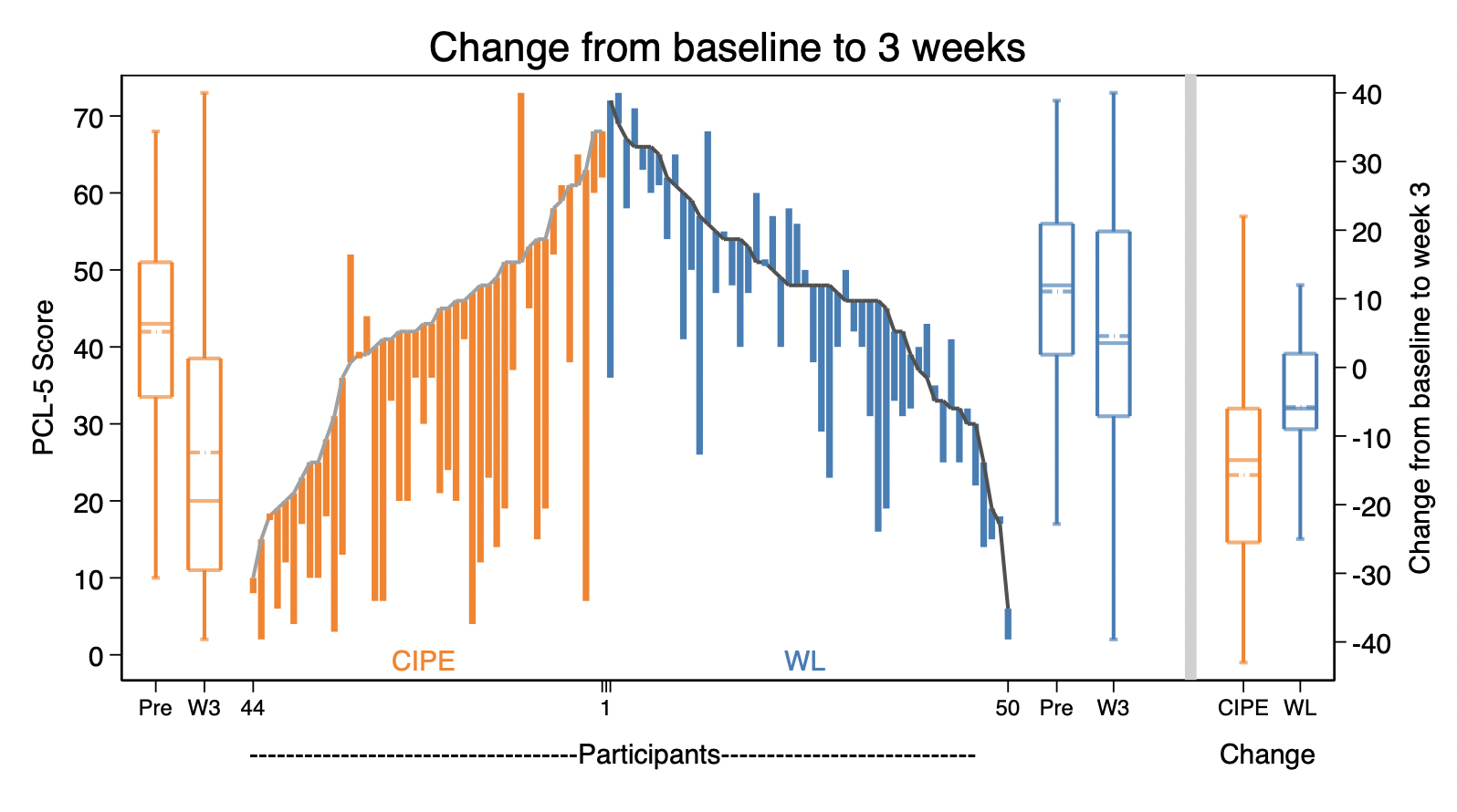
**

**Efig 1**. The distribution of changes in the PTSD Checklist for DSM-5 (PCL-5) sum scores from baseline to end of intervention after three weeks (W3). The parallel line plot is sorted by intervention group and displays the pre- and post-values of each participant, with differences depicted by the length of the line that runs between them. The box plots summarize the pre-, post-, and change scores for each group, with dashed lines that indicate the mean scores.

Abbreviations: CIPE, Condensed Internet-delivered Prolonged Exposure; WL; waiting list; PCL-5, PTSD Checklist for DSM-5.

**Change from baseline to week 7**

**
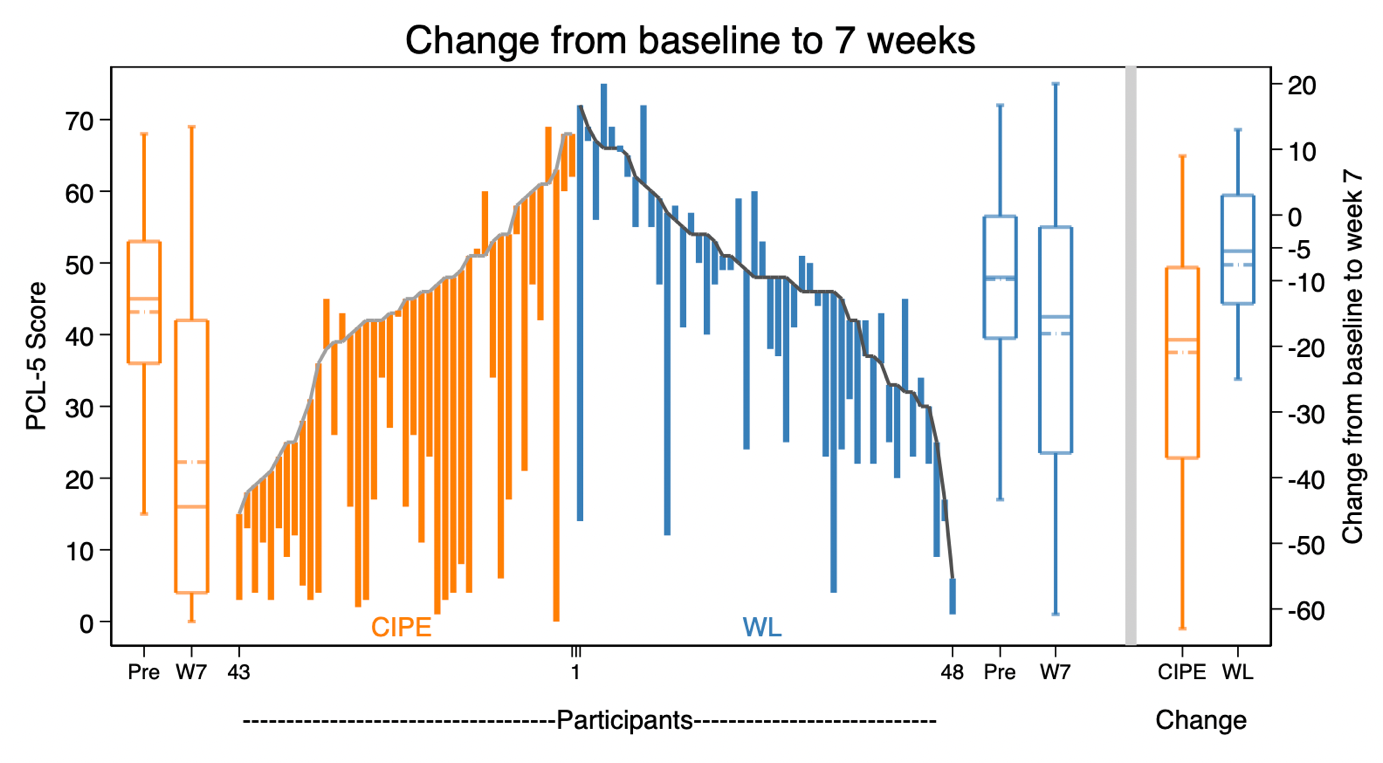
**

**Efig 2.** The distribution of changes in the PTSD Checklist for DSM-5 (PCL-5) sum scores from baseline to week 7 (1-month follow up). The parallel line plot is sorted by intervention group and displays the pre- and post-values of each participant, with differences depicted by the length of the line that runs between them. The box plots summarize the pre-, post-, and change scores for each group, with dashed lines that indicate the mean scores.

Abbreviations: CIPE, Condensed Internet-delivered Prolonged Exposure; WL; waiting list; PCL-5, PTSD Checklist for DSM-5.

**Changes in PTSD symptom clusters**

**eTable 1**. Change in PTSD symptom clusters from baseline

|  | **CIPE** | | **Waiting List** | | **Group x Time** | | | **Effect Size** |
| --- | --- | --- | --- | --- | --- | --- | --- | --- |
| PCL-5 Subscale | M | (SD) | M | (SD) | *β* | *Z* | *P* | Bootstrapped d  (95% CI) |
| **Reexperiencing** |  |  |  |  |  |  |  |  |
| Baseline | 11.67 | (3.87) | 12.62 | (3.76) |  |  |  |  |
| Week 1 | 10.00 | (4.16) | 11.42 | (4.26) |  |  |  |  |
| Week 2 | 8.09 | (4.71) | 10.96 | (4.30) |  |  |  |  |
| Week 3 | 6.97 | (5.26) | 10.52 | (5.04) | 0.95 | 4.22 | <.0001 | 0.64 (0.25 to 1.04) |
| Week 4 | 6.10 | (5.23) | 10.26 | (5.23) |  |  |  |  |
| Week 5 | 5.27 | (4.61) | 9.51 | (5.26) |  |  |  |  |
| Week 6 | 4.14 | (4.67) | 9.62 | (5.10) |  |  |  |  |
| Week 7 | 5.67 | (5.39) | 9.94 | (6.67) | 0.53 | 6.46 | <.0001 | 0.78 (0.38 to 1.18) |
|  |  |  |  |  |  |  |  |  |
| **Avoidance** |  |  |  |  |  |  |  |  |
| Baseline | 5.15 | (1.95) | 5.68 | (2.04) |  |  |  |  |
| Week 1 | 3.93 | (2.14) | 5.53 | (2.15) |  |  |  |  |
| Week 2 | 3.09 | (2.15) | 5.49 | (2.20) |  |  |  |  |
| Week 3 | 2.55 | (2.16) | 5.14 | (2.29) | 0.68 | 5.59 | <.0001 | 0.96 (0.53 to 1.38) |
| Week 4 | 2.11 | (1.90) | 4.63 | (2.53) |  |  |  |  |
| Week 5 | 1.92 | (2.01) | 4.78 | (2.38) |  |  |  |  |
| Week 6 | 1.74 | (2.06) | 5.11 | (2.48) |  |  |  |  |
| Week 7 | 2.16 | (2.32) | 5.04 | (2.48) | 0.30 | 6.89 | <.0001 | 0.93 (0.52 to 1.34) |
|  |  |  |  |  |  |  |  |  |
| **NACM** |  |  |  |  |  |  |  |  |
| Baseline | 12.59 | (6.25) | 13.56 | (5.39) |  |  |  |  |
| Week 1 | 11.80 | (5.78) | 11.80 | (4.98) |  |  |  |  |
| Week 2 | 9.00 | (5.81) | 12.37 | (5.22) |  |  |  |  |
| Week 3 | 7.59 | (6.36) | 12.06 | (55.35) | 1.21 | 4.43 | <.0001 | 0.64 (0.38 to 1.02) |
| Week 4 | 7.07 | (6.52) | 11.67 | (5.55) |  |  |  |  |
| Week 5 | 5.75 | (5.54) | 11.02 | (5.81) |  |  |  |  |
| Week 6 | 5.35 | (5.78) | 11.16 | (5.99) |  |  |  |  |
| Week 7 | 6.49 | (6.47) | 12.02 | (5.79) | 0.71 | 7.41 | <.0001 | 0.83 (0.45 to 1.21) |
|  |  |  |  |  |  |  |  |  |
| **Hyperarousal** |  |  |  |  |  |  |  |  |
| Baseline | 11.59 | (5.06) | 13.31 | (4.72) |  |  |  |  |
| Week 1 | 10.84 | (5.74) | 12.12 | (4.30) |  |  |  |  |
| Week 2 | 9.30 | (5.40) | 12.41 | (5.57) |  |  |  |  |
| Week 3 | 8.14 | (5.98) | 11.94 | (5.21) | 0.75 | 3.24 | <.0001 | 0.43 (0.12 to 0.74) |
| Week 4 | 7.34 | (5.64) | 11.34 | (5.33) |  |  |  |  |
| Week 5 | 6.84 | (5.46) | 10.71 | (5.77) |  |  |  |  |
| Week 6 | 5.91 | (5.07) | 11.04 | (5.93) |  |  |  |  |
| Week 7 | 7.04 | (6.31) | 11.46 | (6.21) | 0.42 | 5.08 | <.0001 | 0.54 (0.17 to 0.90) |

Abbreviations: CIPE, Condensed Internet-Delivered Prolonged Exposure; NACM, Negative Alterations in Cognitions and Mood; PCL-5, PTSD Checklist for DSM-5
